# NAC Transcription Factor *TwNAC01* Positively Regulates Drought Stress Responses in Arabidopsis and Triticale

**DOI:** 10.3389/fpls.2022.877016

**Published:** 2022-06-22

**Authors:** Meng Wang, Li-Tong Ren, Xiao-Yong Wei, Yue-Ming Ling, Hai-Tao Gu, Shan-Shan Wang, Xue-Feng Ma, Guang-Chao Kong

**Affiliations:** ^1^Key Laboratory of Oasis Eco-Agriculture, Xinjiang Production and Construction Corps, Agriculture College of Shihezi University, Shihezi, China; ^2^Institute of Economic Crops, Xinjiang Academy of Agricultural Sciences, Urumqi, China; ^3^School of Chemistry and Chemical Engineering of Shihezi University, Shihezi, China; ^4^Forage Genetics International, West Salem, WI, United States

**Keywords:** gene cloning, gene silencing, NAC transcription factor, Triticale, gene function analyses

## Abstract

The NAC transcription factors play important roles in regulating plant growth, development, and senescence, and responding to biotic and abiotic stressors in plants. A novel coding sequence (1,059 bp) was cloned from hexaploid triticale in this study. The putative protein (352 amino acids) encoded by this sequence was over 95% similar to the amino acid sequence of a NAC protein from *Aegilops tauschii* (XP020161331), and it formed a clade with *Ae. tauschii*, durum wheat, and barley. The putative protein contained a conserved nature actomyosin (NAM) domain (129 consecutive amino acids) between the 20th and 148th amino acids at the N-terminus and three transcription activation regions at the C-terminus. The novel gene was identified as a triticale *NAC* gene localized in the nucleus and designated as *TwNAC01* (GenBank accession MG736919). The expression levels of *TwNAC01* were the highest in roots, followed by leaves and stems when triticale lines were exposed to drought, polyethylene glycol 6,000 (PEG6000), NaCl, cold, methyl jasmonate (MeJA), and abscisic acid (ABA). Transgenic *Arabidopsis thaliana* overexpressing *TwNAC01* had significantly lower leaf water loss rates and longer roots than wild-type (WT) *A. thaliana.* Virus-induced silencing of the *TwNAC01* gene in triticale delayed root development and decreased length of primary root. Under drought stress, leaves of *TwNAC01-*silenced triticale had higher levels of malondialdehyde (MDA) and hydrogen peroxide (H_2_O_2_), but lower relative water content (RWC), net photosynthetic rate, stomatal conductance, intercellular CO_2_ concentration, and transpiration rate than the leaves of the WT. Gene overexpression and silencing experiments suggested that TwNAC01 improves plant stress tolerance by increasing root length, regulating the water content of plant leaves by reducing MDA and H_2_O_2_ content, and adjusting respiration rate. The results suggest that *TwNAC01* is a novel NAC transcription factor gene that can be exploited for triticale and cereal improvement.

## Introduction

Triticale (× *Triticosecale* Wittmack) is a new allopolyploid crop derived from the intergeneric hybridization between wheat (*Triticum*) and rye (*Secale*), followed by chromosome doubling (Zillinsky, 1974). This crop, which can be used as both a food crop and a forage crop, inherits the high grain yield and quality characteristics of wheat with the stress-resistant traits of rye (Sun, 2002; Cao and Kong, 2011). Since triticale exhibits good stress tolerance and it is an important genetic resource for improving wheat and other cereal species, exploring its resistant or tolerant genes against various biotic and abiotic stresses is important for improving not only triticale, but also wheat and other cereals.

During growth and development, plants are often affected by a variety of adverse environmental factors, such as high temperature, drought, salinity, and extreme weather conditions. To overcome these challenges, plants use a series of defense mechanisms against a variety of biotic and abiotic stresses (Cramer, 2010; Pinheiro and Chaves, 2011). By binding to cis-acting elements of the target gene promoter, transcription factors act as molecular switches for gene expression by activating or inhibiting gene expression in response to stress conditions (Nakashima et al., 2012; Puranik et al., 2012). Many transcription factor families, including NAC, WRKY, DREB, and MYB have been found in plants (Puranik et al., 2012). Of these, the plant-specific NAC transcription factor family has the most members (Kim et al., 2004; Pérez-Rodríguez et al., 2010). Several studies have shown that NAC transcription factors play important roles in a variety of plant processes, including growth and development, leaf senescence, hormone increase and decrease, and regulation of defense response to biotic and abiotic stresses (Nakashima et al., 2012; Puranik et al., 2012). For example, in *Arabidopsis thaliana,* overexpression of the *AtNAC2* gene improved lateral root elongation (He et al., 2005). In addition, Jensen et al. (2013) reported that *Arabidopsis* overexpressing *ATAF1 s*howed obvious dwarfism and flowering delay; the endogenous ABA content of the transgenic *Arabidopsis* overexpressing *ATAF1* was also significantly greater (a 6–8-fold increase) than that of the wild-type (WT) counterpart, which improved the drought tolerance of the transgenic lines. Similarly, overexpression of the *OsNAC10* gene, which is specifically expressed in rice roots, increased the diameter of rice roots, improved drought tolerance of rice, and significantly increased rice yield under drought stress (Jeong et al., 2010). Also in rice, overexpression of the *SNAC1* gene improved drought and salt tolerance in field and greenhouse; transgenic plants overexpressing *SNAC1* also had lower water loss rates and were more sensitive to ABA stress than WT plants (Xiong et al., 2001). Overexpression of the wheat genes *TaNAC2* and *TaNAC67* in *A. thaliana* significantly improved tolerance to salt, drought and cold stress (Mao et al., 2012). Indeed, RT-PCR analysis showed that *TaNAC4* and *TaNAC8* were induced by phytohormones (e.g., salicylic acid, SA; methyl jasmonate, MeJA; and abscisic acid, ABA), pathogens (stripe rust), abiotic stressors (salinity and cold), and mechanical injury (Kang et al., 2010). Transgenic wheat overexpressing the *TaNAC69* gene had greater biomass and longer roots than the control, and thus had a better survival rate under salt and drought stresses (Xue et al., 2011). These studies demonstrated that *NAC* genes can be induced by biotic and abiotic stresses under a variety of conditions, and thus are likely to play critical roles in stress tolerance in plant. However, little is known about *NAC* genes in triticale. To address this knowledge gap, we aimed to identify and characterize *NAC* genes in triticale.

In this study, a novel *NAC* transcription factor gene, *TwNAC01*, from triticale was cloned. Gene expression characteristics demonstrated that *TwNAC01* responded to various abiotic stresses. We characterized the gene in detail for further exploration of triticale and cereal improvement.

## Materials and Methods

### Plant Materials

A drought tolerant triticale cultivar., “Xinxiaoheimai 3” (developed and kindly provided by the Wheat Crop Research Institute of Shihezi University, Xinjiang, China; Li et al., 2008) was used for this study. After cleaning and disinfection, the seeds were planted in an arid lot with irrigation at the Experimental Station of the Agricultural College of Shihezi University (85° 59′ 50″ N, 44° 18′ 58″ E, Xinjiang, China). The experiment station is located at an altitude about 437–450 m with an annual rainfall of 208 mm, on average, and an evaporation capacity of about 1,660 mm, a typical continental climate suitable for irrigated agriculture. The soil type was calcaric fluvisol, a sandy loam soil texture with 19.5% clay, 32.1% silt, and 38.5% sand. The soil bulk (0–20-cm depth) property was characterized as 1.34 g cm^−3^ in density with 15.3 g kg^−1^ organic matter, 1.0 g kg^−1^ total *N*, 19.3 mg kg^−1^ Olsen P and 194 mg kg^−1^ total K.

To eliminate the effects of rainfall, a rain shelter was built over the drought stress plots. Plants flowering at the same time were selected during the flowering stage. In drought-stressed plots, irrigation was stopped when the triticale developed at the heading stage, and the control was irrigated normally. The plots were laid out randomly with three replications in both irrigated and drought-stressed areas. Proline, MDA and electrical conductivity levels in the plant leaves were assessed daily as described previously (Chen and Wang, 2002). Roots, stems, flag leaves, and immature grains of both the stressed and control plants were collected at a point when the proline, MDA and electrical conductivity increased (67.30%, 42.12%, and 37.67%, respectively) significantly, and the soil moisture content decreased (37.32%) significantly in the stressed plots.

### Full-Length 5′- and 3′-Race

RNA was extracted from the above-mentioned triticale leaves using Hipure HP Plant RNA Mini Kits (Magen). Using these RNA as templates, we performed reverse transcriptase PCR (RT-PCR) to synthesize cDNA sequences using SMARTScribe genome Reverse Transcriptase (TaKaRa, Dalian, China). Synthesized cDNA was stored at −20°C before being sequenced. Based on RNA-Seq sequencing results, the sequence of Unigene c51971 (708 bp) was studied in this research, and specific primers for 5′- and 3′-RACE using SMARTer RACE kits (Supplementary Table S1) were designed. The RACE procedure was as follows: 94°C for 2 min, 94°C for 30 s, 55°C for 30 s, 72°C for 1 min, 72°C for 35 min, and 16°C for 10 min. The PCR products were recovered and purified using 1.0% agarose gel electrophoresis. The purified PCR products were ligated to the pMD19-T vector (TaKaRa, Dalian, China) and transformed into TOPO10 cells (TIANGEN, China). Positive clones were identified and sequenced. The open reading frame (ORF) of the full-length cDNA sequence was obtained by splicing the sequencing results using an online tool CAP3^1^ (Shang et al., 2019) and the National Center for Biotechnology Information Basic Local Alignment Search Tool (NCBI-BLAST). Sequence alignment analysis preliminarily identified the sequence as a triticale *NAC* gene, which was preliminarily designated *TwNAC01*.

### Analysis of the Triticale *TwNAC01* Sequence

The NCBI ORF Finder^2^ was used to identify sequences homologous to the ORF and coding sequences of *TwNAC01* (GenBank: MG736919). Conserved structures in the TwNAC01 gene were identified using Smart^3^ and the ProtParam tool.^4^ Physical and chemical properties of the predicted TwNAC01 protein were analyzed, as well as its hydrophobicity^5^ using the ExPASy server.^6^ Multi-alignment of TwNAC01 with other NAC proteins in different species was conducted using DNAMAN. Subcellular location of the protein was predicted using Protcomp and TargetP 1.1.^7^ The relevant sequences were aligned using MEGA (version 10.0; Shang et al., 2019) and MegAlign (DNAStar).

### Real-Time Fluorescence Quantitative PCR

The RNA samples used for real-time fluorescence quantitative PCR (qRT-PCR) were from two sources, the drought-stressed triticale plants from stressed plots as mentioned above in plant materials, and seedlings treated with 20% PEG6000 (osmotic potential of about −0.50 MPa; Michel and Kaufmann, 1973; Michel et al., 1983), 200-mM NaCl, cold (4°C), 100-μM MeJA, or 100-μM ABA as follows (Huang et al., 2015). The triticale seedlings were cultured at 25°C with a normal watering regime, under a 12-h light/12-h dark cycle in a growth chamber. The seedlings were transplanted in hydroponic boxes when they had two leaves and one main shoot. Three hydroponic boxes were allocated to each treatment; each box had 12 holes with 5 seedlings in each hole. After transplantation, seedlings were allowed to acclimate for 5 days. After acclimation, one set of boxes was transferred to a cold room (4°C). Other boxes were treated with each of the followings: 1 L of 20% PEG6000 (osmotic potential of about −0.50 MPa; Michel and Kaufmann, 1973; Michel et al., 1983), 200-mM NaCl, 100-μM MeJA, and 100-μM ABA, respectively. In all treatments, plant roots were soaked and leaves were sprayed with the same solution. Roots and leaves were collected after 0, 1, 3, 6, 12, and 24 h of treatment. Collected samples were frozen in liquid nitrogen and then transferred to a freezer at −80°C. The relative expression levels of target genes were calculated with the 2^−ΔΔCt^ method (Livak and Schmittgen, 2001).

RNA was extracted from the triticale materials collected from each of the six treatments (field drought, 20% PEG6000, 200-mM NaCl, cold (4°C), 100-μM MeJA, and 100-μM ABA) using Hipure HP Plant RNA Mini Kits (Magen, China), and cDNA was synthesized using 5X All-ln-One RT MasterMix (Applied Biological Materials, Canada) with specific primers (qRT-PCR primer pair in Supplementary Table S1). The wheat actin gene, *TaActin*, was used as internal reference for real-time qPCR (Supplementary Table S1). qPCR was performed using SuperReal PreMixPlus (SYBR Green) kits (Tiangen, China). Each 10-μl qPCR volume contained 6 μl 2× SuperReal PreMixPlus, 0.25-μl forward primer, 0.25-μl reverse primer, 1-μl cDNA template, and sufficient ddH_2_O to make 10 μl. qRT-PCR amplifications were performed using a Roche Light-Cycler 480R with the following cycling conditions: pre-denaturation at 95°C for 15 min, followed by 40 cycles of denaturation at 95°C for 10 s, annealing at 61°C for 30 s, and amplification at 72°C for 30 s. All reactions were performed in triplicate, and relative gene expression levels were determined using the 2^-ΔΔCt^ method (Livak and Schmittgen, 2001).

### Subcellular Localization of the TwNAC01 Protein

The coding sequence of the *TwNAC01* gene was cloned into the plant subcellular expression vector pCAMBIA1301S Enhanced Green Fluorescent Protein (EGFP; GenBank accession E17099). Insertion primers containing *Bam*HI-*Xba*I restriction sites (Supplementary Table S1) were designed and ligated using a ClonExpressII one-step cloning kit (TaKaRa, Dalian, China). After verification *via* sequencing, the recombinant plasmid and the empty vector control (VC) were transformed into *Agrobacterium tumefaciens* GV3101 (TaKaRa, Dalian, China; Mao et al., 2014). GV3101 carrying the recombinant plasmid or the empty vector (TaKaRa, Dalian, China) was cultured on Luria-Bertani (LB) medium containing Kan + and Rif+. When the OD600 of the bacterial solution reached 0.5–0.6, the bacterial solution was collected and re-suspended in infection buffer [10-mM MgCl_2_, 10-mM fatty acid methyl ester sulfonate (MES), 150-μM surfactant-AS, pH 5.7].

Subcellular location was visualized in tobacco leaves. Tobacco seeds were planted in a growth chamber and cultured at 23°C, with 60% relative humidity and a 16-h light/8-h dark cycle, for 3 weeks prior to vector inoculation. The cultured *A. tumefaciens* solution was then injected into tobacco leaves with 5-ml needleless sterile syringe, and tobacco seedlings were then cultured in darkness for 36 h. Tobacco leaves exhibiting normal growth after inoculation were selected for examination. The area of each selected leaf around the infection site was excised. Enhanced Green Fluorescent Protein (EGFP) fluorescence signals in the tobacco leaves were observed using a Fluo-View confocal microscope (FV300, Olympus, Japan).

### Generation of Transgenic *Arabidopsis* Overexpressing *TwNAC01*

To obtain transgenic *Arabidopsis* plants, the coding sequence containing the stop codon of *TwNAC01* was amplified by RT-PCR and cloned into the *Kpn*I and *Xba*I restriction sites of the pCAMBIA1300-35S vector (TaKaRa, Dalian, China) under the control of the 35S promoter of the cauliflower mosaic virus (CMV). The primers containing the *Kpn*I and *Xba*I restriction sites are listed in Supplementary Table S1. The recombinant vector pCAMBIA1300-35S-TwNAC01 and the empty vector pCAMBIA1300-35S-VC were introduced into *A. tumefaciens* strain GV3101 (TaKaRa, Dalian, China). Finally, transgenic *Arabidopsis* plants were generated using the *A. tumefaciens*-mediated floral dipping method (Clough and Bent, 1998). To generate homozygous progeny, T1 and T2 seeds were selected on kanamycin (50 mg l^−1^) plates. T3 transgenic and WT plants of *A. thaliana* were watered once at the rosette stage and then subjected to drought stress for 25 days. RNA was then extracted from the leaves and roots of both transgenic and WT *A. thaliana* using Hipure HP Plant RNA Mini Kits (Magen, China). *TwNAC01* gene expression levels were then measured using semi-quantitative analysis with gene-specific primers (Supplementary Table S1) following the same qPCR protocol. Representative lines overexpressing *TwNAC01* were used for further analysis.

### Drought Tolerance of Transgenic *Arabidopsis thaliana* Overexpressing *TwNAC01*

We then measured various stress-related physiological indexes in 35-day-old transgenic *A. thaliana* overexpressing *TwNAC01*, mock-transformed *A. thaliana*, and WT *A. thaliana.* Leaf relative water content (RWC) was determined following the methods of Flexas et al. (2006), leaf electrical conductivity was measured following Chen and Wang (2002), leaf MDA content was estimated following Chen and Wang (2002), and leaf H_2_O_2_ content was determined using an H_2_O_2_ measurement kit (China Nanjing Jiancheng Science and Technology Co., Ltd). The concentration of hydrogen peroxide was estimated according to a standard formulae as: H_2_O_2_ (mmol/gprot) = (OD_tissue_ − OD_blank_)/(OD_standard_ − OD_blank_) * standard concentration (163 mmol/l), where H_2_O_2_ (mmol/gprot) is the hydrogen peroxide concentration measured from the testing tissue, OD_tissue_ is the OD value measured from the testing tissue, OD_blank_ is the OD value measured from the blank control, and OD_standard_ is the OD value of the kit standard. We also determined the rate of water loss in leaves. Five rosette leaves from each group of *A. thaliana* plants (WT, VC, and the three *TwNAC01-*overexpression lines) were collected, transferred to filter paper, and placed in a constant temperature incubator at 25°C. Leaves were weighed every hour for 8 h and photographed at three time points (2, 5, and 8 h). Water loss was characterized based on weight loss and the degree of leaf curl. Water loss rate measurements were replicated six times. After growing for 55 days, plants of all *A. thaliana* lines were carefully removed from the nutrient soil and washed. The length of the main root system of each plant was measured.

### Virus-Induced Gene Silencing of the Triticale *TwNAC01* Gene

Specific primers were designed for PCR amplification of silencing fragments based on the 3′-UTR region of the *TwNAC01* gene (Supplementary Table S1), and the barley stripe mosaic virus (BSMV) vector was constructed using ligation-independent cloning (LIC) as previously described (Lee et al., 2015). The BSMV-*γb* vector was digested with the *Apa*I restriction enzyme and the vector skeleton was recovered. The PCR fragments were then treated with T4 DNA polymerase in a reaction mixture containing 1-mM deoxythymidine triphosphate (dTTP). The mixture was subjected to react for 30 min at room temperature. After the completion of the reaction, the system was heated to 75°C for 10 min to inactivate the T4 DNA polymerase. The treated fragments (200 ng) and the BSMV vector (20 ng) were mixed, heated to 66°C for 2 min, and then cooled slowly to room temperature. We transformed 10 μl of the mixture into *Escherichia coli* using the heat shock method. Positive clones were screened using colony PCR and verified *via* sequencing. The positive clones were shaken, and the plasmids were extracted for follow-up experiments. The extracted viral vector plasmids were transferred into *A. tumefaciens* GV3101 for triticale inoculation.

### Stress Tolerance of Triticale After *TwNAC01* Gene Silencing

*A. tumefaciens* carrying BSMV-phytoene desaturase BSMV::asTaPDS constructs (BSMV-*PDS*) induce photobleaching or yellow–orange coloration in the silenced tissue due to depletion of enzymes involved in biosynthesis of carotenoid pigments or chlorophyll, respectively (Lee et al., 2015). Thus, these constructs may be used as positive controls for gene silencing. Triticale plants were inoculated with BSMV-*PDS*, BSMV-*γb*, or BSMV-*TwNAC01* for about a week (two to three leaves were treated per plant). After an additional 2 weeks of growth, white stripes began to appear on the leaves due to the expression of the indicator gene. At this point, samples of the leaves were taken and stored at −80°C. Total RNA was extracted from these samples for quantitative reverse transcription PCR (qRT-PCR). The RWC of the leaves was determined following the methods of Flexas et al. (2006), MDA content in the leaves was estimated following Chen and Wang (2002), and H_2_O_2_ content in the leaves was determined using an H_2_O_2_ measurement kit (Nanjing Jiancheng Science and Technology Co., Ltd). Stomatal conductance, net photosynthesis rate, transpiration rate, and intercellular CO_2_ concentration were measured using a LI-6400 portable photosynthesis meter (Li-Cor). All these physiological parameters were determined in four replications, respectively.

### Statistical Analysis

Microsoft Excel was used for data analysis, and one-way ANOVA was conducted using SPSS Statistics 22.0 software to assess significance of differences. The data were analyzed using Student’s *t*-test and the difference was considered significant statistically at *p* < 0.05.

## Results

### The Full-Length *TwNAC01* Gene

Using primers designed based on the sequence of Unigene c51971 (GSP-R/F in Supplementary Table S1), we amplified an intermediate sequence from the extracted triticale RNA (Supplementary Figure S1A) that was 502-bp long (Supplementary Figure S1B). Based on this intermediate sequence, we used 5′-RACE to amplify the 224 bp 5′ sequence (Supplementary Figure S1C), and 3′-RACE to amplify the 557 bp 3′ sequence (Supplementary Figure S1D). Splicing of the 5′ and 3′ sequences based on the intermediate sequence (Unigene c51971) yielded a fill-length ORF of 1,059 bp. Using specific primers designed out of this ORF sequence, we successfully amplified the gene (Supplementary Figure S1E). Transformation of this gene fragment into *E. coli via* the pMD19-T vector confirmed the expression of a 1,059-bp sequence (Supplementary Figure S1F). The predicted amino acid sequence of the gene was 352-bp long and had over 95% homology with the NAC amino acid sequences from barley, wheat, and other plants. We thus inferred that the cloned gene was a triticale *NAC* gene, which was designated *TwNAC01*. This gene has been submitted to GenBank (accession number MG736919).

### Gene Sequence Analysis

The molecular formula of the encoded protein was predicted to be C_1722_H_2642_N_464_O_522_S_19_, with a predicted molecular weight of 38805.86 kDa, and a theoretical isoelectric point of 5.44. The total number of positive/negative charge residues predicted in this protein were 46/37, and the atomic composition was C_1722_, H_2642_, N_464_, O_522_, and S_19_. The extinction coefficient of the predicted protein was 46,996, and its absorbance at a wavelength of 280 nm was 1.211 l g^−1^ cm^−1^. The total average hydrophobic coefficient of the predicted protein was −0.494, indicating that the putative protein was hydrophilic.

**Figure 1:**
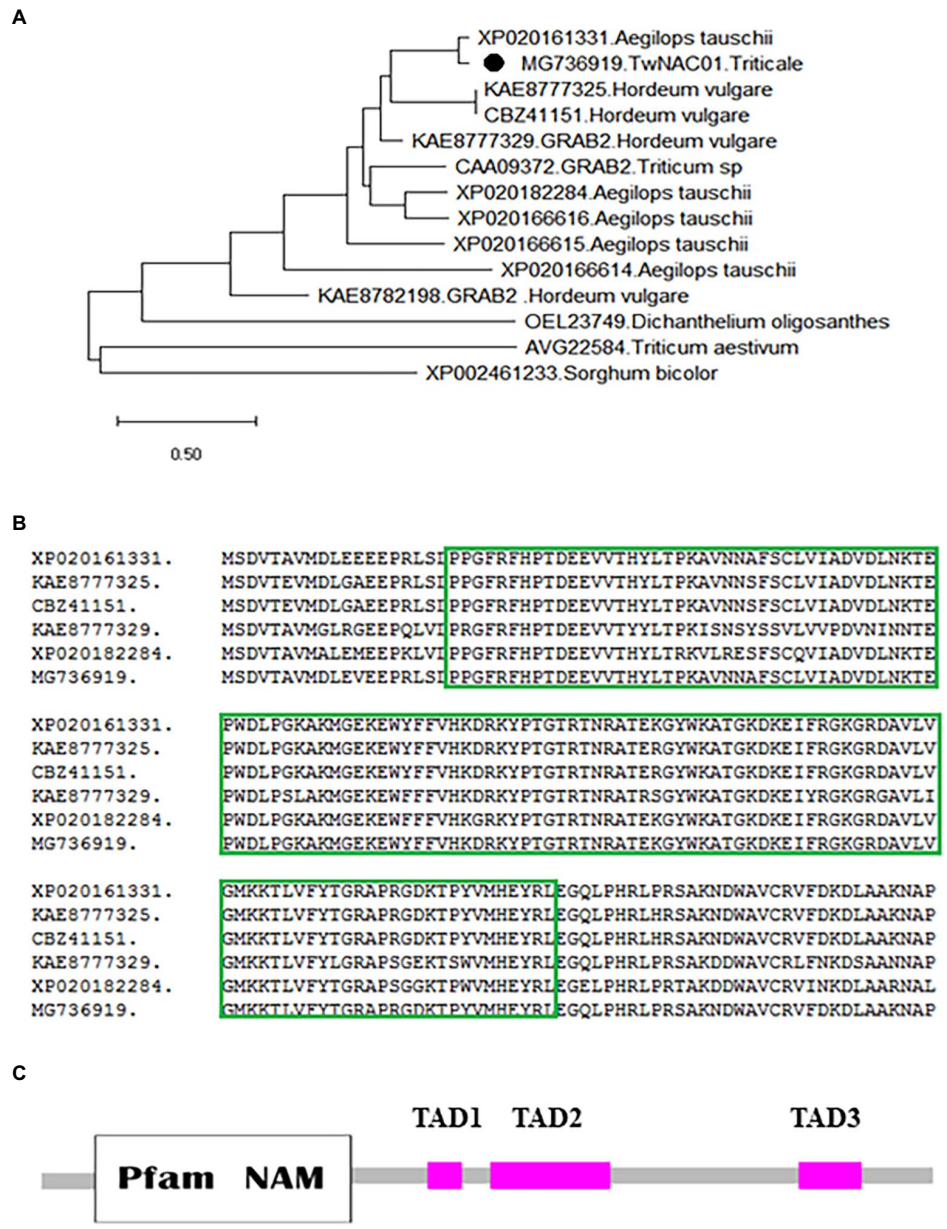
Phylogenetic relationships among NAC proteins in plants, and the conserved NAM superfamily of *TwNAC01*. **(A)** Neighbor-joining phylogenetic relationships between the TwNAC01 and other NAC proteins in plants; **(B)** Amino acid sequence alignment analysis showing the conserved homeodomain regions of *TwNAC01* with its closest homologs from barley, wheat, *Aegilopsis tauschii*, and durum wheat; **(C)** The conserved NAM superfamily domain in the TwNAC01 protein. TAD, transcription activation domain.

A NJ phylogenetic tree based on sequence similarity showed that the triticale TwNAC01 protein formed a clade with NAC proteins from *Aegilops tauschii* (XP-020161331), and *Hordeum vulgare* (KAE8777325 and CBZ41151; Figure 1A). In particular, the amino acid sequence of TwNAC01 was more than 97% similar to *Ae. tauschii* protein AtNAC92 (XP20161331.1; Figure 1B). The predicted TwNAC01 sequence contained a conserved NAM-superfamily domain composed of 129 consecutive amino acids at the N-terminus (between amino acid 20 and 148) and three transcriptional activation domains at the C-terminus (Figure 1C).

### Subcellular Localization in the Nucleus

**Figure 2:**
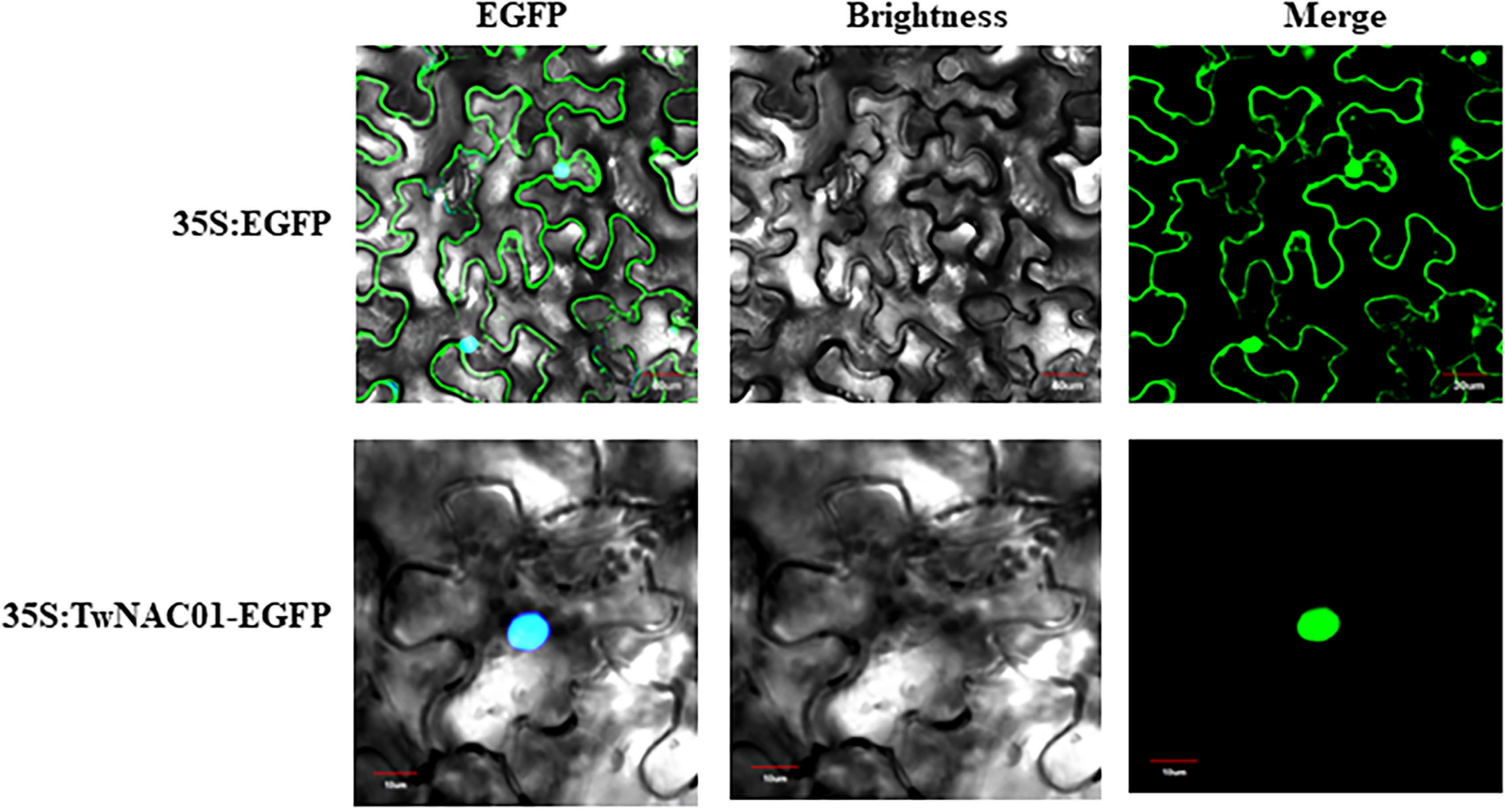
Subcellular localization of the TwNAC01 protein in the nuclei of tobacco cells.

Localization prediction analysis indicated that the TwNAC01 protein was not located in the chloroplasts or mitochondria. This protein was unlikely to be a chloroplast transport peptide, mitochondrial transport peptide, or signal peptide. The target protein was found in other organelles secretory pathways. Further study with the Protcomp analysis indicated that the predicted protein was located in the nucleus. The shear site was the 62th amino acids with the maximum value of 0.113, and the comprehensive splicing site was the 62th amino acids with the maximum value of 0.107 (No signal peptide; Table 1). The shear site was consistent with the predicted upper transmembrane region and signal peptide (Table 1). In the control group (35S:EGFP), the EGFP localization signal was dispersed throughout the cell, with the strongest signals originating primarily from the cell membrane and nucleus (Figure 2). In the treatment group (35S:TwNAC01-EGFP), the EGFP protein signal was restricted to the nucleus (Figure 2). This suggested that the fusion protein was located in the nucleus, as was predicted by our bioinformatics analysis.

**Table 1:** Subcellular localization scores.

^a^Position	cTP	mTP	SP	Other	Loc	RC	TPlen
Positioning score	0.09	0.104	0.171	0.887	_	2	_
^b^Position	Nucleus	Cell membrane	Extracellular	Mitochondria	Chloroplast	Bubble	
Positioning score	8.26	1.01	0.04	0.01	0.11	0.48	

a *cTP, chloroplast transit peptide; mTP, mitochondrial transit peptide; SP, signal peptide; RC, reliability level; Tplen, Other organelles*.

b *Scores were obtained online using the Target P1.1 Server (http://www.cbs.dtu.dk/services/TargetP/)*.

### Analysis of *TwNAC01* Gene Expression in Triticale Under Drought Stress

**Figure 3:**
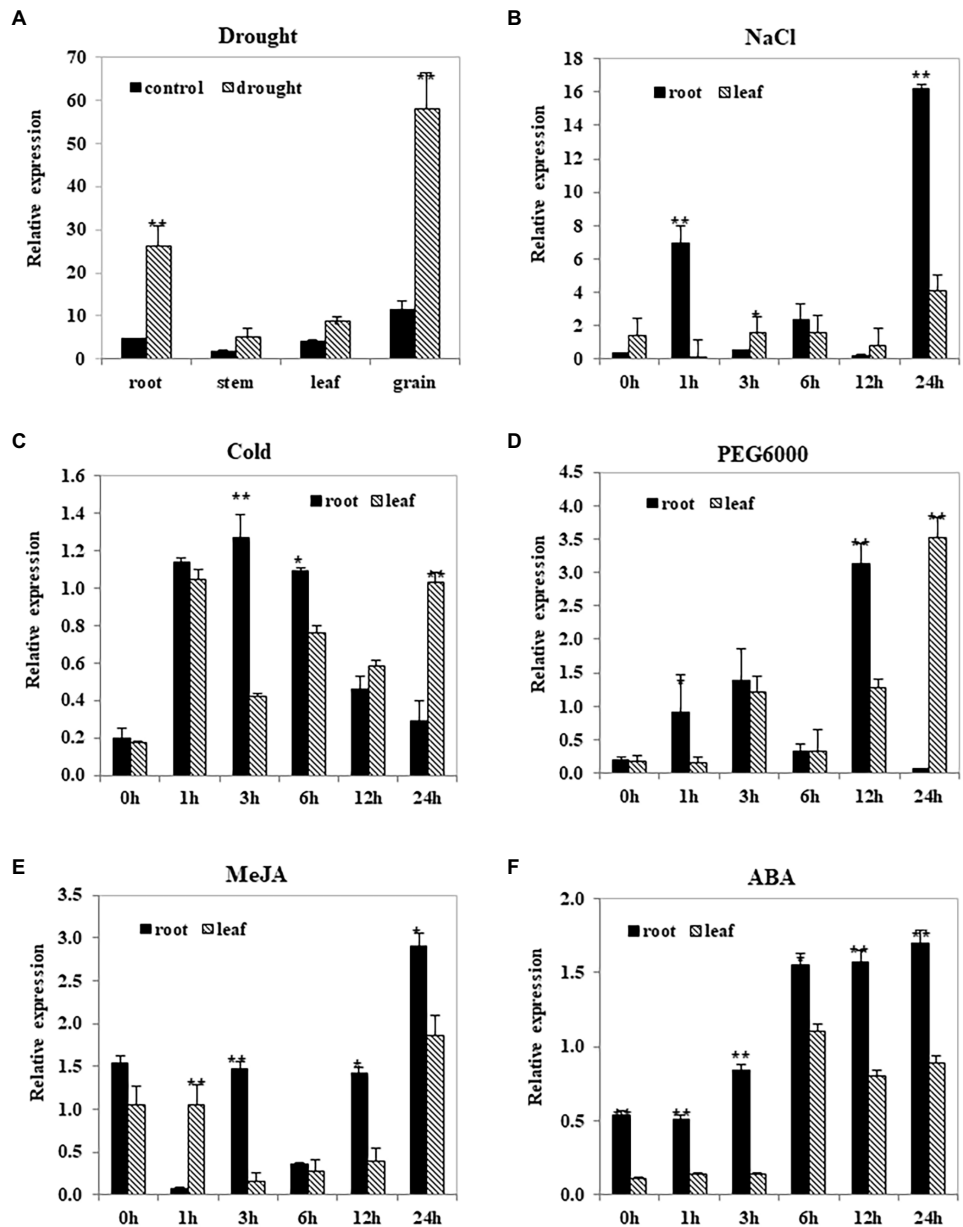
*TwNAC01* gene expression patterns in triticale after stress treatments. Triticale plants were subjected to one of the following treatments: **(A)** drought, **(B)** 200-mM NaCl, **(C)** cold (4°C), **(D)** 20% PEG6000, **(E)** 100-μM MeJA, or **(F)** 100-μM ABA. Relative expression levels in the plant tissues were determined using qRT-PCR and the 2^−ΔΔCT^ method. Transcript levels were normalized to the wheat actin genes (*TaActin*). Values shown are the means ± SE of three replicates of three independent samples. ^*^ and ^**^ indicates significant difference at 0.05% and 0.01% level, respectively.

The *TwNAC01* gene was significantly upregulated (*p* < 0.01) in triticale roots and grains of drought-stressed plants as compared to the control (Figure 3A); *TwNAC01* was also upregulated with respect to the control in the leaves (*p* < 0.05), but upregulated insignificantly with respect to the control in the stems. This suggested that the triticale *TwNAC01* gene was upregulated in response to drought stress, with the strongest upregulation found in the grain, followed by the root, leaf, and stem.

After 1- and 24-h NaCl treatment, *TwNAC01* expression was significantly more upregulated in the roots than in the leaves (Figure 3B). In contrast, after 24 h of cold and PEG6000-induced dehydration stress, *TwNAC01* was significantly upregulated in the leaves as compared to the roots (although *TwNAC01* was significantly upregulated in the roots as compared to the leaves after 12-h PEG6000 treatment; Figures 3C,D). After 24 h of MeJA and ABA stress, *TwNAC01* was significantly upregulated in the roots as compared to the leaves; in response to these stressors, *TwNAC01* expression levels appeared to increase over time (Figures 3E,F). After ABA treatment, *TwNAC01* gene expression levels in roots were significantly higher than those in leaves throughout the time course (Figure 3F). Overall, the data indicated that the *TwNAC01* gene played a role in the stress response of the triticale roots and leaves.

### Confirmation of Transgenic *Arabidopsis thaliana* Lines Overexpressing *TwNAC01*

PCR analysis of *E. coli* transformed with the pCAMBIA1300-35S overexpression vector recovered a 1,059-bp band, indicating that transformation had been successful and that the target gene was ligated to the vector (Supplementary Figure S2A). After restriction endonuclease digestion, the vector skeleton (10 kb) and a band slightly longer than 1,000 bp were obtained (Supplementary Figure S2B), again indicating that the recombinant expression plasmid containing the target gene were successfully constructed. The *A. tumefaciens* solution containing the expression vector plasmid was also analyzed using PCR, and a 1,059-bp band was confirmed (Supplementary Figure S2C). After the plasmid was introduced into *A. thaliana* inflorescences using the floral dip method, three T_0_ plants expressing *TwNAC01* were identified *via* 1/2 MS Kan + medium followed by PCR verification. Derived from these T_0_ lines, three T_3_ lines were confirmed to express *TwNAC01*.

### Root Length and Leaf Water Loss Rate in Transgenic *Arabidopsis* Overexpressing *TwNAC01*

**Figure 4:**
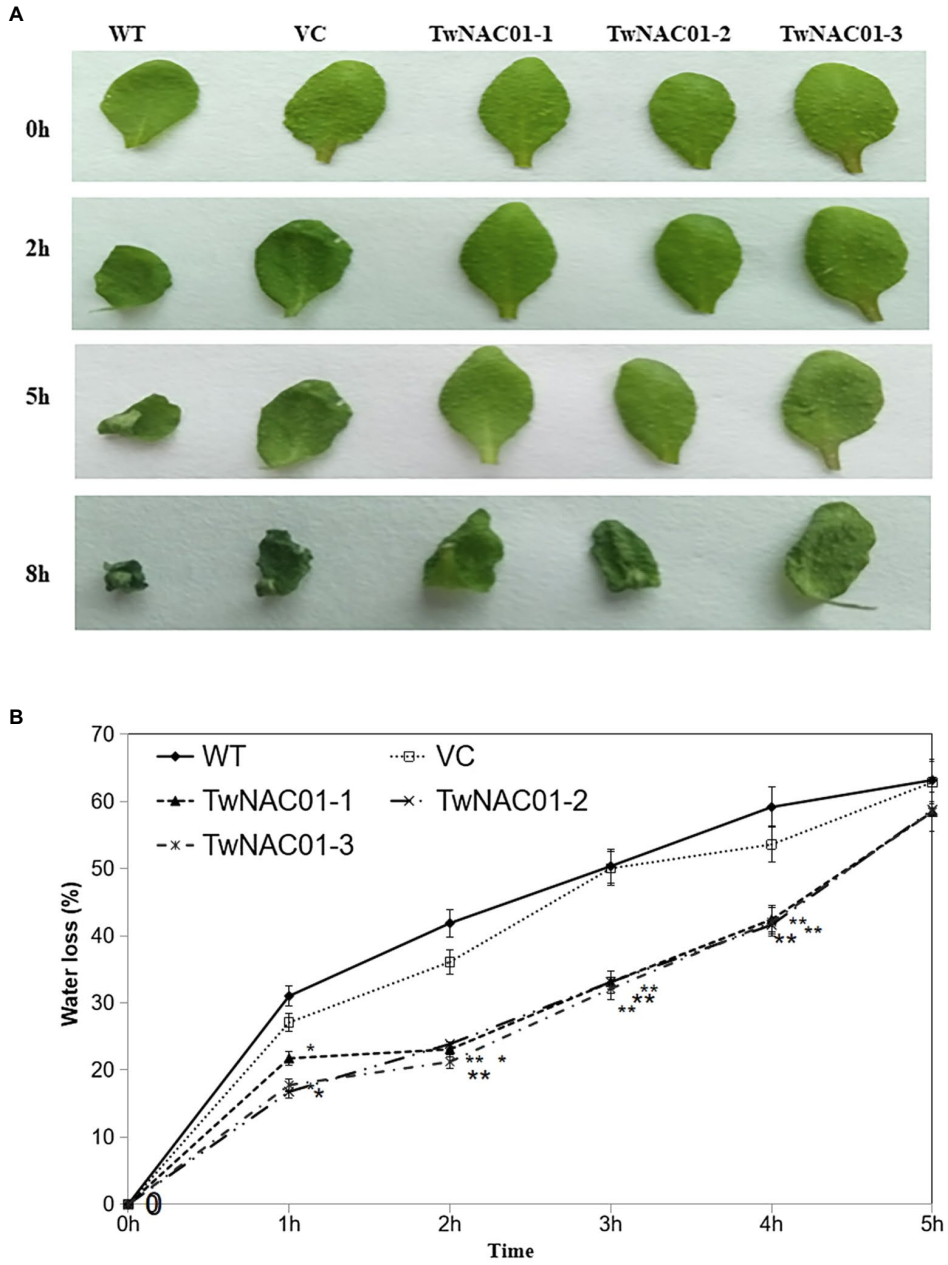
Three lines overexpressing *TwNAC01* (TwNAC01-1, TwNAC01-2, and TwNAC01-3) in Arabidopsis exhibit reduced rates of water loss in the leaves compared to WT (wild type) and VC (vector control). **(A)** Leaves of various Arabidopsis lines after 0–8 h of dehydration; **(B)** Rates of moisture loss in the leaves of various Arabidopsis lines. Values are means ± SE of three replicates. ^*^ and ^**^indicates significant difference at 0.05% and 0.01% level, respectively.

After 2 h of treatment at 25°C, while the leaves of *A. thaliana* WT and those expressing the VC had curled slightly, the leaves of the transgenic *A. thaliana* lines overexpressing *TwNAC01* (TwNAC01-1, TwNAC01-2, and TwNAC01-3) exhibited no obvious curling (Figure 4A). After 5 h at 25°C, the leaves of the WT and VC plants were obviously curled, while the leaves of the *TwNAC01*-overexpressing lines were only slightly curled. After 8 h of dehydration, the leaves of the WT and VC plants were noticeably withered and crumpled in appearance; although the leaves of the transgenic lines were also somewhat withered, the observed degree of dehydration was much less severe (Figure 4A).

**Figure 5:**
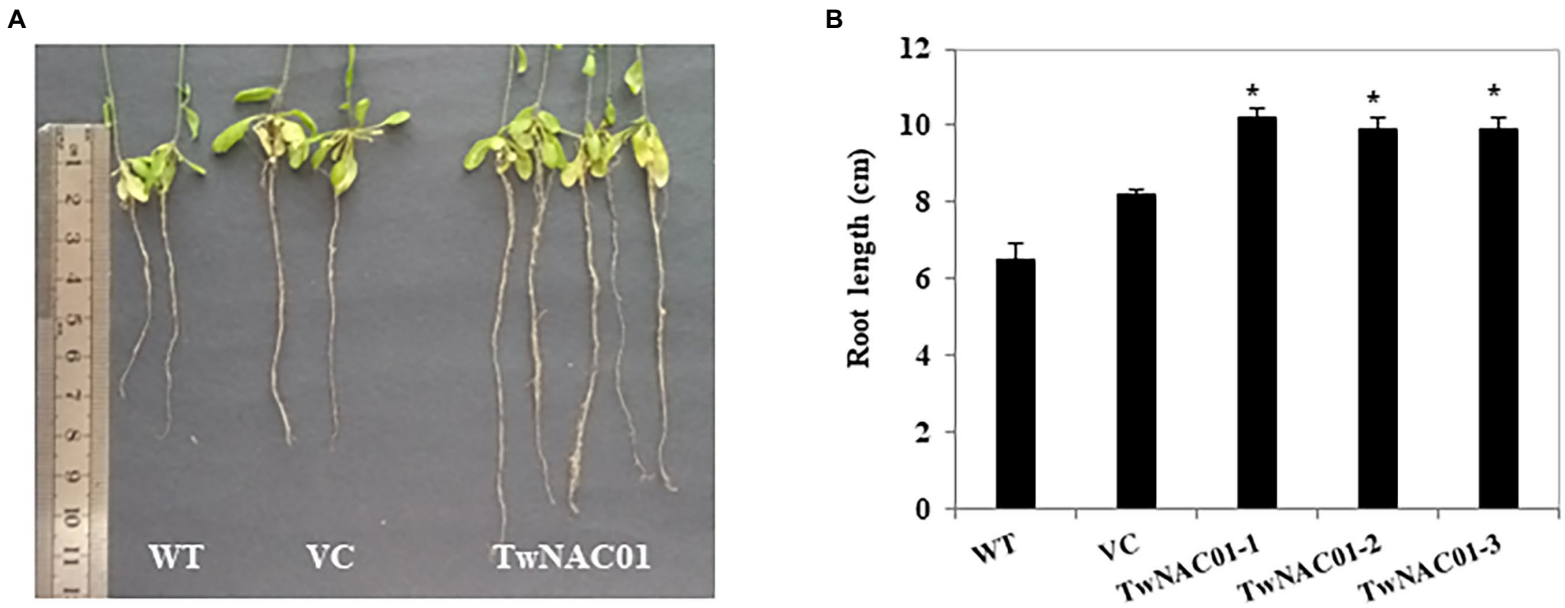
Root growth of the WT (wild type), VC (vector control), and transgenic Arabidopsis lines overexpressing *TwNAC01*. **(A)** Primary root length of WT, VC, and transgenic plants grown for 55 d; **(B)** Statistical analysis of the root growth. Values shown are the means ± SE of three replicates of three independent samples. ^*^ and ^**^indicates significant difference at 0.05% and 0.01% level, respectively.

However, the three transgenics slowed down water loss (29.90%–49.30%) significantly, especially during the first 2 h. As drying continued, water loss rates increased almost linearly in all lines (Figure 4B). The overall data indicated that water loss rates in the transgenic lines were significantly (*p* < 0.05) lower (16.77%–58.77%) than those in the WT (31.02%–68.18%) and VC (27.07%–62.86%) lines (Figure 4B). This result should attribute to the longer roots (9.90 ± 0.03–10.20 ± 0.24 cm) in transgenic lines relative to the WT (6.52 ± 0.41 cm) and VC (8.18 ± 0.17 cm) counterparts (differences of 1.5-fold and 1.2-fold, respectively; Figures 5A,B).

### Physiological Indexes of Stress Tolerance in *Arabidopsis thaliana* Overexpressing *TwNAC01*

**Figure 6:**
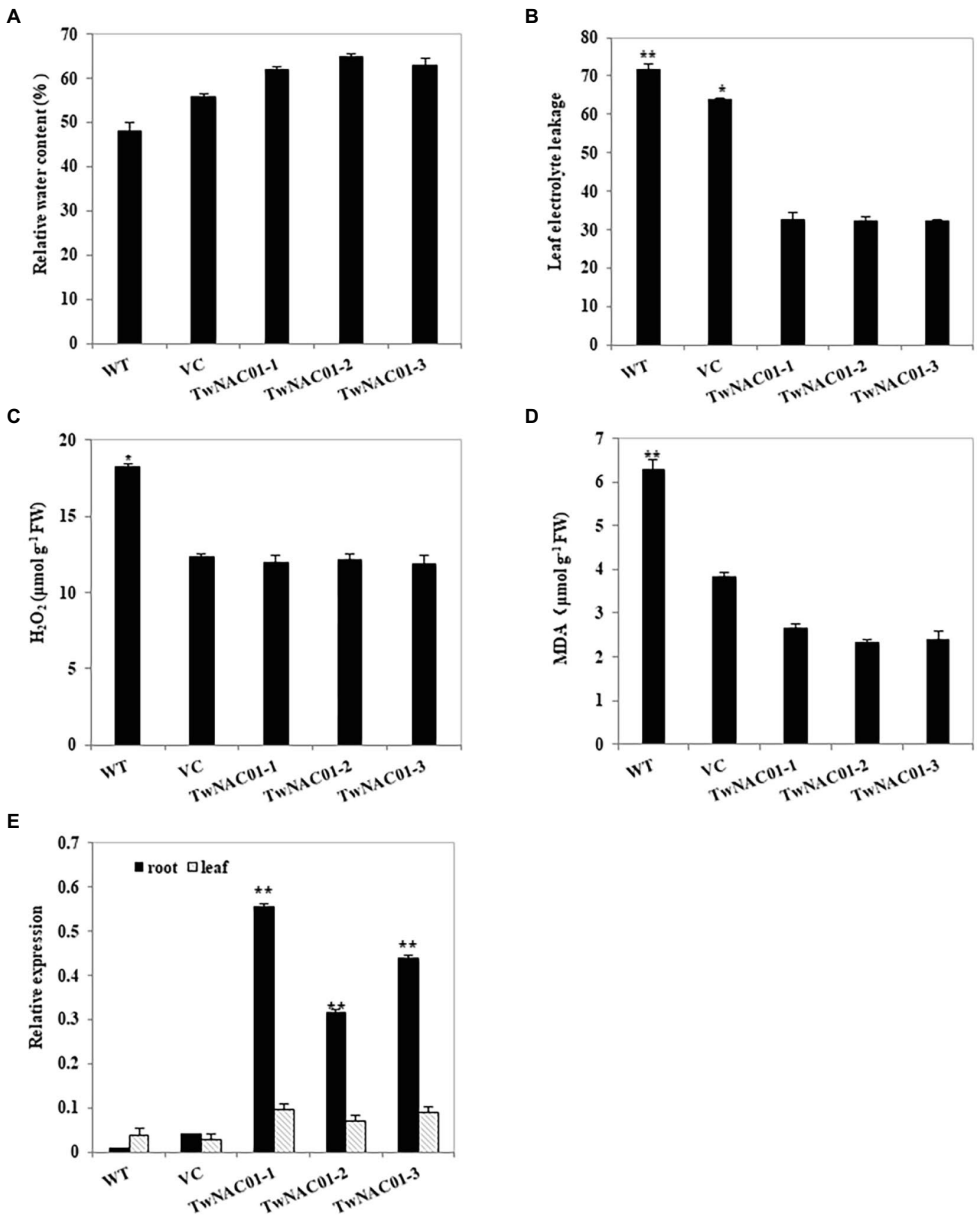
Analysis of physiological indexes of the transgenic Arabidopsis lines overexpressing *TwNAC01* under drought stress conditions. Analyses of leaf relative water content (%) **(A)** leaf electrolyte leakage **(B)**, H_2_O_2_ content (μmol g^−1^ FW; **C**), and MDA content (μmol g^−1^ FW; **D**) in WT (wild type) and transgenic lines overexpressing *TwNAC01* lines under drought-stressed conditions. Relative expression of *TwNAC01* in the roots and leaves of transgenic *Arabidopsis thaliana* lines **(E)**. Values are means ± SE of three replicates. ^*^ and ^**^indicates significant difference at 0.05% and 0.01% level, respectively.

Although the relative leaf water contents (62.03%–64.70%) of the three transgenic *Arabidopsis* lines were slightly higher than those of VC (55.92%) and WT (48.09%) lines, these differences were not significant (Figure 6A). However, leaf electrical conductivity (reflecting electrolyte leakage and thus membrane damage) was significantly greater in the VC (63.67%) and WT (71.60%) lines as compared to the transgenic lines (32.07%–32.60%; Figure 6B). Hydrogen peroxide (18.29 μmol g^−1^ FW) and MDA (6.27 μmol g^−1^ FW) levels were significantly greater in the leaves of the WT line as compared to all other lines; there were no significant differences in H_2_O_2_ or MDA levels between the VC plants (14.37 μmol g^−1^ FW and 5.81 μmol g^−1^ FW) and any of the transgenic lines (11.85–12.12 μmol g^−1^ FW and 2.32–2.66 μmol g^−1^ FW; Figures 6C,D).

After drought stress, *TwNAC01* was upregulated in the roots and leaves of the transgenic plants as compared to the VC and WT plants; in all three transgenic lines, *TwNAC01* gene expression was significantly greater in the roots than it in the leaves (Figure 6E). On average, *TwNAC01* gene expression levels in the transgenic *A. thaliana* lines were 8-fold and 38-fold greater than in the VC and WT plants, respectively. Thus, in response to drought stress, *TwNAC01* was upregulated in transgenic *A. thaliana* overexpressing *TwNAC01* as compared to mock-transformed and WT controls.

### Expression of *TwNAC01* After Virus-Induced Gene Silencing in Triticale Under Drought Stress

**Figure 7:**
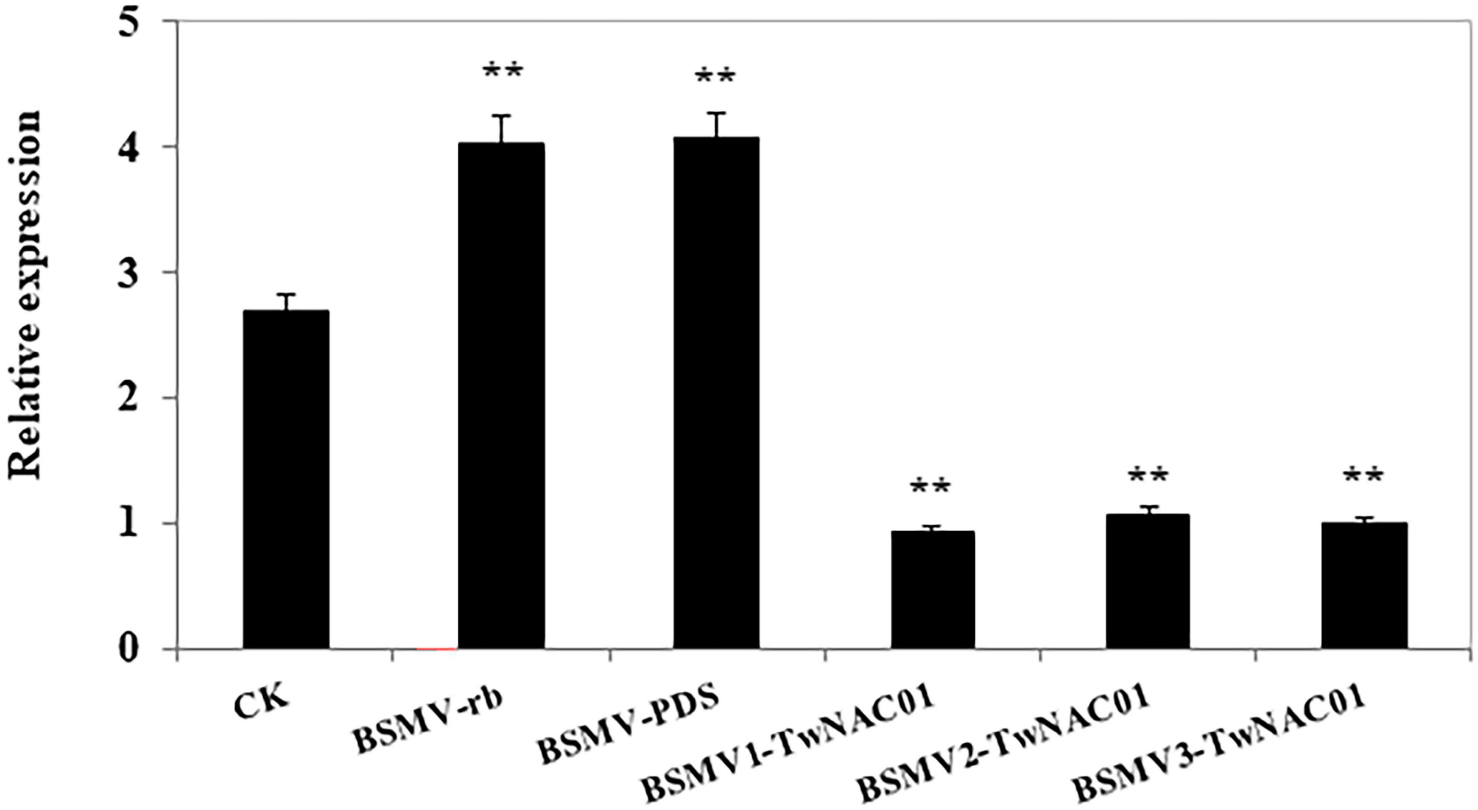
Relative *TwNAC01* expression patterns in triticale after silencing the *TwNAC01* gene, as determined using qRT-PCR and the 2^−ΔΔCT^ method. Transcript levels were normalized to *TaActin*. CK, wide type control; BSMV-*γb*, empty vector control; BSMV-*PDS*, BSMV with indicator gene; BSMV-*TwNAC01*, vector carrying silenced *TwNAC01*. Values shown are the means ± SE of three replicates of three independent samples. ^**^indicates significant difference at 0.01% level.

A 327-bp sequence was amplified from the triticale cDNA as mentioned above using the virus-induced gene silencing (VIGS) primers (Supplementary Figure S3A). After transformation of the amplified sequence into *E. coli*, positive clones were identified *via* PCR amplification (Supplementary Figures S3B,C). After drought stress, *TwNAC01* gene expression levels in the control were significantly greater than those in the *TwNAC01-*silenced plants (BSMV-*TwNAC01*; Figure 7). In contrast, drought stress significantly upregulated *TwNAC01* in the empty vector (BSMV-*γb*) and indicator-gene (BSMV-*PDS*) plants as compared to the control. This indicated that drought stress upregulated the triticale *TwNAC01* gene; *TwNAC01* was downregulated significantly even under drought conditions when it was silenced (Figure 7).

### Phenotypic Implications of *TwNAC01* Gene Silencing in Triticale

**Figure 8:**
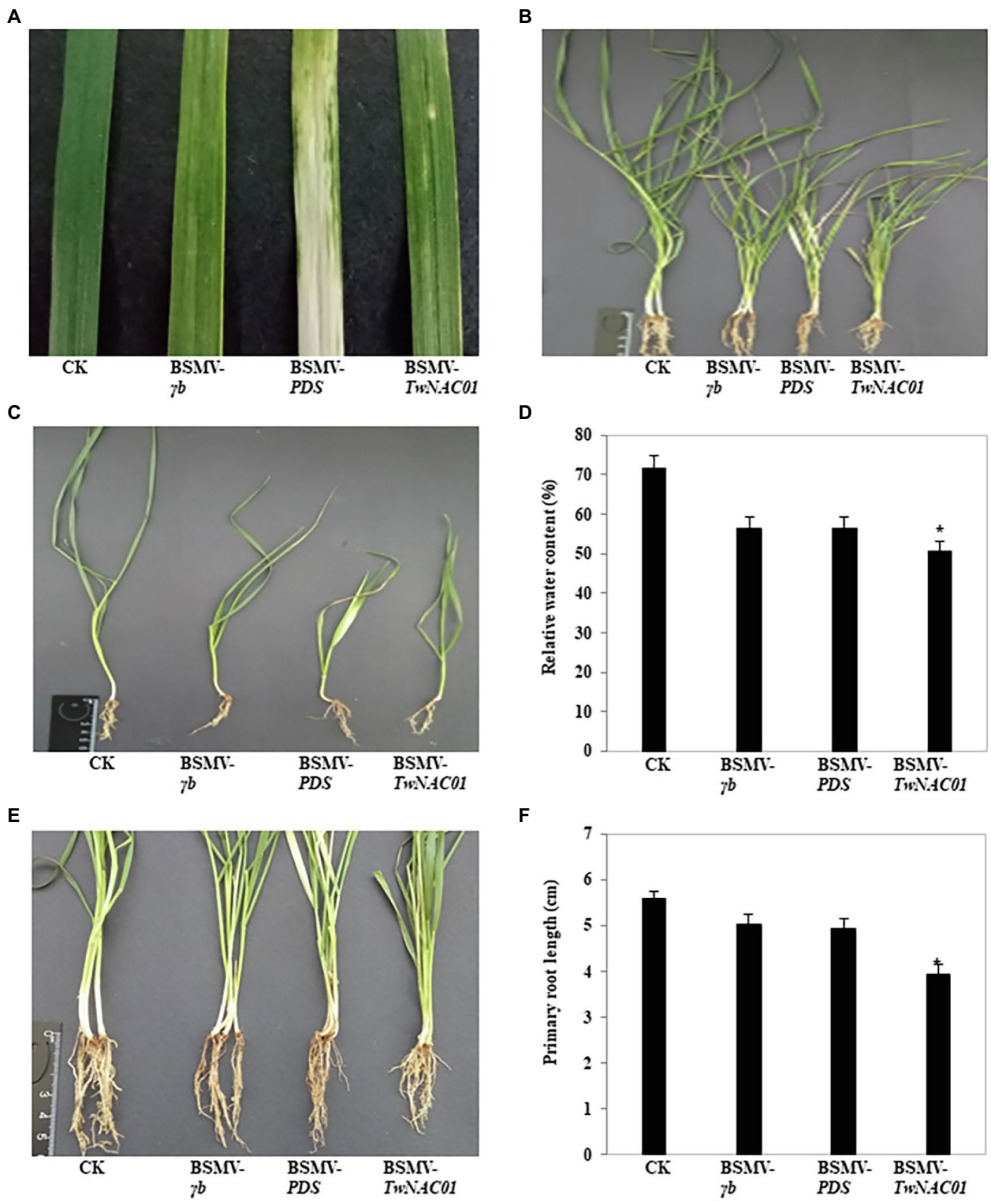
Phenotypic differences among triticale plants inoculated with different BSMV vectors for the VIGS of *TwNAC01.* **(A)** Leaves showing viral infection; **(B,C)** Biomass comparison; **(D)** Relative water contents (%); **(E)** Visual comparison of root lengths; **(F)** Root length quantification. Values shown are means ± SE of three replicates. ^*^indicates significant difference at 0.05% level. CK, control; BSMV-*γb*, empty vector; BSMV-*PDS*, BSMV with indicator gene; BSMV-*TwNAC01*, vector carrying silenced *TwNAC01*.

About 2 weeks after inoculation with the BSMV vectors, the indicator-gene (BSMV-*PDS*) plants began to exhibit symptoms of stripe mosaic virus. On the 20th day after inoculation, large areas of the leaves of the BSMV-*PDS* plants were bleached, while the leaves of empty vector (BSMV-*γb*) and *TwNAC01*-silenced (BSMV-TwNAC01) plants showed slight bleaching (Figure 8A). In general, the growth potential of each of the inoculated groups (BSMV-*γb*, BSMV-*PDS*, and BSMV-*TwNAC01*) was weaker than that of the control (Figures 8B,C). The mean RWC (57.64%) of the leaves of BSMV-*TwNAC01* was significantly lower than that of the leaves of the control (71.63%; Figure 8D). The roots of the *TwNAC01*-silenced plants were significantly shorter (3.95 cm) than those of the control (5.58 cm), BSMV-*γb* (5.04 cm), and indicator-gene plants (4.95 cm; Figures 8E,F). The results indicated that *TwNAC01* gene silencing inhibits triticale root development and significantly reduced triticale growth.

### Physiological Indexes of Drought Stress and Photosynthesis in *TwNAC01*-Silenced Triticale

**Figure 9:**
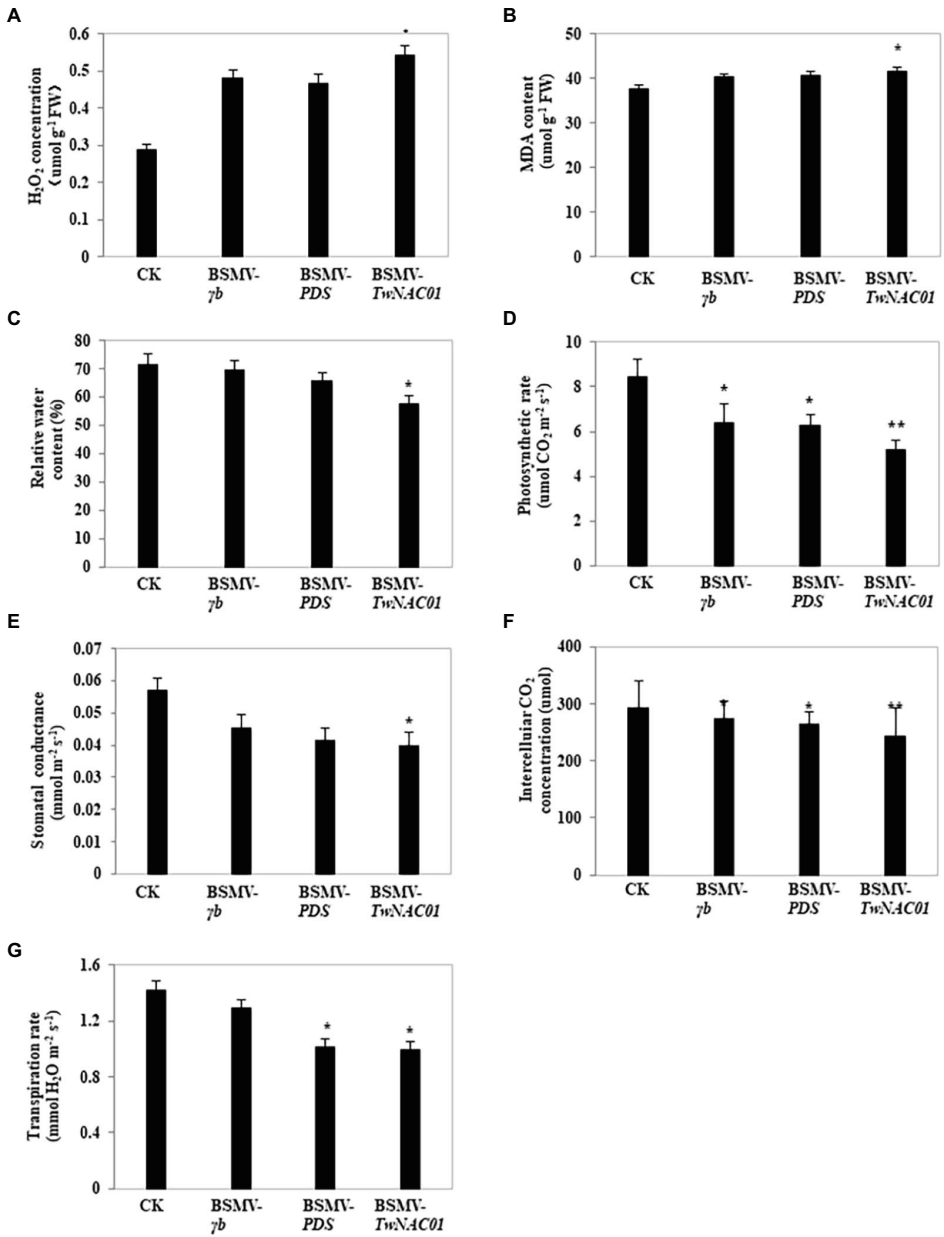
Physiological indexes of drought stress and photosynthesis in triticale leaves after virus-induced gene silencing using BSMV. **(A)** H_2_O_2_ concentration (μmol g^−1^ FW); **(B)** MDA content (μmol g^−1^ FW); **(C)** Relative water content (RWC; %); **(D)** Photosynthetic rate (μmol CO_2_ m^−2^ s^−1^); **(E)** Stomatal conductance to H_2_O (mmol m^−2^ s^−1^); **(F)** Intercellular CO_2_ concentration (μmol); **(G)** Transpiration rate (mmol H_2_O m^−2^ s^−1^). Values shown are means ± SE of three replicates. ^*^ and ^**^, indicates significant difference at *p* < 0.05, 0.01, respectively. CK, control; BSMV-*γb*, empty vector; BSMV-*PDS*, BSMV with indicator gene; BSMV-*TwNAC01*, vector carrying silenced *TwNAC01*.

After drought stress, levels of H_2_O_2_ (54.17 μmol g^−1^ FW) and MDA (41.49 μmol g^−1^ FW) in the leaves of BSMV-*TwNAC01* plants were significantly higher (Figures 9A,B), while RWC was significantly lower (57.64%; Figure 9C) than those in the leaves of uninfected control plants (65.51%–71.63%). This result demonstrated that the triticale leaves were more stressed by drought when the *TwNAC01* gene was silenced, i.e., the stress-resistant ability of the plant decreased after silencing the *TwNAC01* gene. The data suggested that *TwNAC01* played an important role in coping with stress. Furthermore, net photosynthetic rate, stomatal conductance to H_2_O, intracellular CO_2_ level, and transpiration rate were significantly lower in the BSMV-*TwNAC01* leaves as compared to the control (Figures 9D–G). Net photosynthetic rate and intracellular CO_2_ level were significantly lower than the control in the BSMV-*γb* and BSMV-*PDS* leaves (Figures 9D,F), while transpiration rate was significantly lower than the control in the BSMV-*PDS* leaves (Figure 9G).

## Discussion

NAC transcription factors are considered the most important family of transcription factors in plants; these transcription factors play various important roles in stress response, as well as in the regulation of plant growth and development (Xu et al., 2015). Stress-related NAC transcription factors have been well examined in wheat, rice, and *Leymus triticoides*. Here, we used RACE RNA-seq and RT-PCR to clone the first putative *NAC* gene from hexaploid triticale. This putative *NAC* gene had over 95% similarity with known *NAC* genes from other crops, including common wheat, *Aegilops*, and durum wheat. These findings were consistent with our phylogenetic analysis. In addition, the predicted protein sequence of the putative *NAC* gene included a conserved NAM domain between the 20th and 148th amino acids at the N-terminus, as well as three transcriptional activation regions at the C-terminus. We thus concluded that the putative gene was indeed an *NAC* gene. This gene was designated *TwNAC01.*

Our results showed that *TwNAC01* was upregulated by a variety of abiotic stressors and signal molecules, including salinity, drought, PEG6000, and ABA. Several studies have shown that about 20%–25% of plant *NAC* genes respond to stress treatments and participate in stress alleviation (Fang et al., 2008; Nuruzzaman et al., 2010; Puranik et al., 2012). In addition, *A. thaliana* overexpressing the wheat *NAC* genes *TaNAC2* and *TaNAC67* exhibited significantly improved tolerance to drought, salinity, and cold as compared to the WT control (Mao et al., 2014). Similarly, overexpression of the wheat *NAC* genes (*TaNAC2a, TaNAC4a, TaNAC6, TaNAC7, TaNAC13,* and *TaNTL5*) in tobacco significantly improved drought tolerance (Tang et al., 2012). Previous studies have also shown that *NAC* gene expression increases in response to drought stress (Nakashima et al., 2007; Wu et al., 2009; Chen et al., 2014). In this study of triticale, *TwNAC01* expression was significantly upregulated in the drought-stressed plant roots and young grains as compared to unstressed controls. This suggested that *TwNAC01* expression is induced by drought stress, and it plays an important role in response to drought in triticale. *NAC* genes have also been shown to be upregulated in aging plant tissues, by treatment with plant signal molecules (e.g., ABA, ethephon, JA, and SA; Bu et al., 2008; Jensen et al., 2010; Xia et al., 2010; Tang et al., 2012), and by exposure to ethylene and methyl jasmonate (Scharrenberg et al., 2003). The upregulation of *TwNAC01* in response to stress was stronger in roots than in leaves, which was in agreement with a previous study in wheat showing that *TaNAC4* was more strongly upregulated in wheat roots compared to leaves and stems (Jensen et al., 2007; Xia et al., 2010). The data suggest that *TwNAC01* is upregulated in triticale roots first in response to stress, in order to promote the development of plant roots and reduce the damage caused by adverse conditions.

The mechanisms by which *NAC* transcription factors alleviate drought stress in plants have been well studied. For example, overexpression of the *NAC* gene *ATAF1* in *A. thaliana* decreased transpiration rate and increased drought tolerance (Christiansen et al., 2011). Similarly, rice overexpressing *OsNAP* had lower water loss rates during vegetative growth, increased sensitivity to exogenous ABA, and improved tolerance to salt, drought, and low temperature stress (Negi et al., 2018). Also in rice, NAC transcription factors regulate the expression of *OsSRO1c* genes, which are primarily expressed in guard cells; the overexpression of *OsSRO1c* increases H_2_O_2_ accumulation in guard cells and reduces the number of completely open stomata, thus reducing water loss *via* transpiration (You et al., 2013). Finally, the *SNAC3* gene in rice targets a ROS-scavenging gene, and *SNAC3* overexpression upregulates this target gene (Fang et al., 2015). Previous studies suggest that, when plants are under stress, the NAC family act as transcriptional activators or repressors of their downstream genes, such as NAC regulatory network interactions with jasmonic acid-, salicylic acid- and ethylene-mediated stress responses *via* both ABA-dependent and independent pathways by binding to the promoter of Early Response to Dehydration 1 (ERD1; Figueroa et al., 2021). These regulatory networks can reduce transpiration rate by promoting plant root elongation, reducing H_2_O_2_ and MDA accumulation, and increasing leaf water content. Ultimately, these factors enhance plant adaptability to adversity. In this study, *TwNAC01* expression was successfully silenced in triticale BSMV-*TwNAC01* plants: *TwNAC01* expression levels in the triticale BSMV-*TwNAC01* plants were significantly lower than those in the control, BSMV-*γb*, and BSMV-*PDS* plants. In general, the growth potential of the *TwNAC01-*silenced plants was significantly lower than that of the other lines. In particular, the *TwNAC01-*silenced plants had shorter roots and reduced water content as compared to the other plants. Under drought conditions, indexes of physiological stress (i.e., MDA and H_2_O_2_ levels) were significantly increased in *TwNAC01-*silenced plants as compared to the controls, while relative water content and indexes of photosynthetic activity (net photosynthetic rate, stomatal conductance, transpiration rate, and intercellular CO_2_ concentration) were significantly reduced. The observed changes in these physiological indexes suggested that triticale growth and stress tolerance were substantially impaired by *TwNAC01* silencing.

## Conclusion

In conclusion, overexpression of the triticale *TwNAC01* gene in *A. thaliana* improved drought tolerance of *A. thaliana* by increasing the water retention capacity of leaves, reducing cellular membrane damage, decreasing production of ROS in the leaves, and promoting root elongation. In *TwNAC01*-silenced triticale, leaf relative water content and root length were significantly decreased as compared to the control, while leaf H_2_O_2_ and MDA levels were significantly increased. Leaf net photosynthetic rate, stomatal conductance, intercellular CO_2_ concentration, and transpiration rate were also significantly lower in the *TwNAC01*-silenced plants as compared to the control. These results indicated that *TwNAC01* silencing decreased drought tolerance of triticale, suggesting that the *TwNAC01* gene plays an important role in response to drought stress in triticale.

## Data Availability Statement

The datasets presented in this study can be found in online repositories. The names of the repository/repositories and accession number(s) can be found at: https://www.ncbi.nlm.nih.gov/nuccore/1545818062.

## Author Contributions

G-CK planned and designed the research and wrote the main manuscript text. MW performed most of the experiments and data acquisition and participated in figure preparation and manuscript organization. L-TR, X-YW, H-TG, S-SW, and Y-ML helped with experiments and data analysis. X-FM reviewed and revised the manuscript. All authors contributed to the article and approved the submitted version.

## Funding

This study was supported by the National Natural Science Foundation of China (31860376 and 31360333) and the Ministry of Agriculture 948 project (2013-Z75).

## Conflict of Interest

X-FM was employed by the company Forage Genetics International, West Salem, WI, United States.

The remaining authors declare that the research was conducted in the absence of any commercial or financial relationships that could be construed as a potential conflict of interest.

## Publisher’s Note

All claims expressed in this article are solely those of the authors and do not necessarily represent those of their affiliated organizations, or those of the publisher, the editors and the reviewers. Any product that may be evaluated in this article, or claim that may be made by its manufacturer, is not guaranteed or endorsed by the publisher.

## Footnotes

^1^ http://doua.prabi.fr/software/cap3

^2^ https://www.ncbi.nlm.nih.gov/orffinder/

^3^ http://smart.embl-heidelberg.de/

^4^ https://web.expasy.org/protparam/

^5^ https://web.expasy.org/cgi-bin/protparam/protparam

^6^ https://web.expasy.org

^7^ http://www.cbs.dtu.dk/services/TargetP
