# Supplementary material for: NAC Transcription Factor TwNAC01 Positively Regulates Drought Stress Responses in Arabidopsis and Triticale
Source: Front Plant Sci. 2022 Jun 22;13:877016. doi: 10.3389/fpls.2022.877016 (PMC9257188; doi:10.3389/fpls.2022.877016)
Supplement: Supplementary file 1 [file Data_Sheet_1.docx]

**Supplementary materials**

**Table S1** Primer sequences used in this study.

| Use | Primer name | Sequence (5' to 3′) |
| --- | --- | --- |
| RT-PCR | RT-R/F | AGTCCTTGTCGAACACCCGG  CGTCGGCATGAAGAAGACGC |
| TaActin | TaActin-R/F | TACAGTGTCTGGATCGGTGGT  GGAAAAGTGCAGAGAGACACG |
| UniGene sequence | GSP-R/F | GGCAAACAGCCCAATCGT  ATGTCGGACGTGACGGC |
| 3' RACE | C065-1  C065-2 | TGGGGGACCAGCAGACCGCGATC  TCGTCGGCGCTGCTGAGCCCTTC |
| 5′ RACE | B086-1  B086-2  B086-3 | CGGTCTTGTTGAGGTC  CTTGGGGGTGAGGTAGT  GGTGGAACCGGAAGCC |
| Gene length | TwNAC01-R/F | ATGTCGGACGTGACGGCGGTG  TCAGATCTTCCACATGTTGG |
| Subcellular localization | EGFP-R/F | ACTCTAGACATGTCGGACGTGACGGCGGT  GACGGATCCTCAGATCTTCCACATGTTGGAGTAGTCC |
| Silent fragment | BSMVNAC-R/F | AAGGAAGTTTAACCCAAGGCCGTCAACAAC  AACCACCACCACCGTCGGTACTCGTGCATGACG |

**
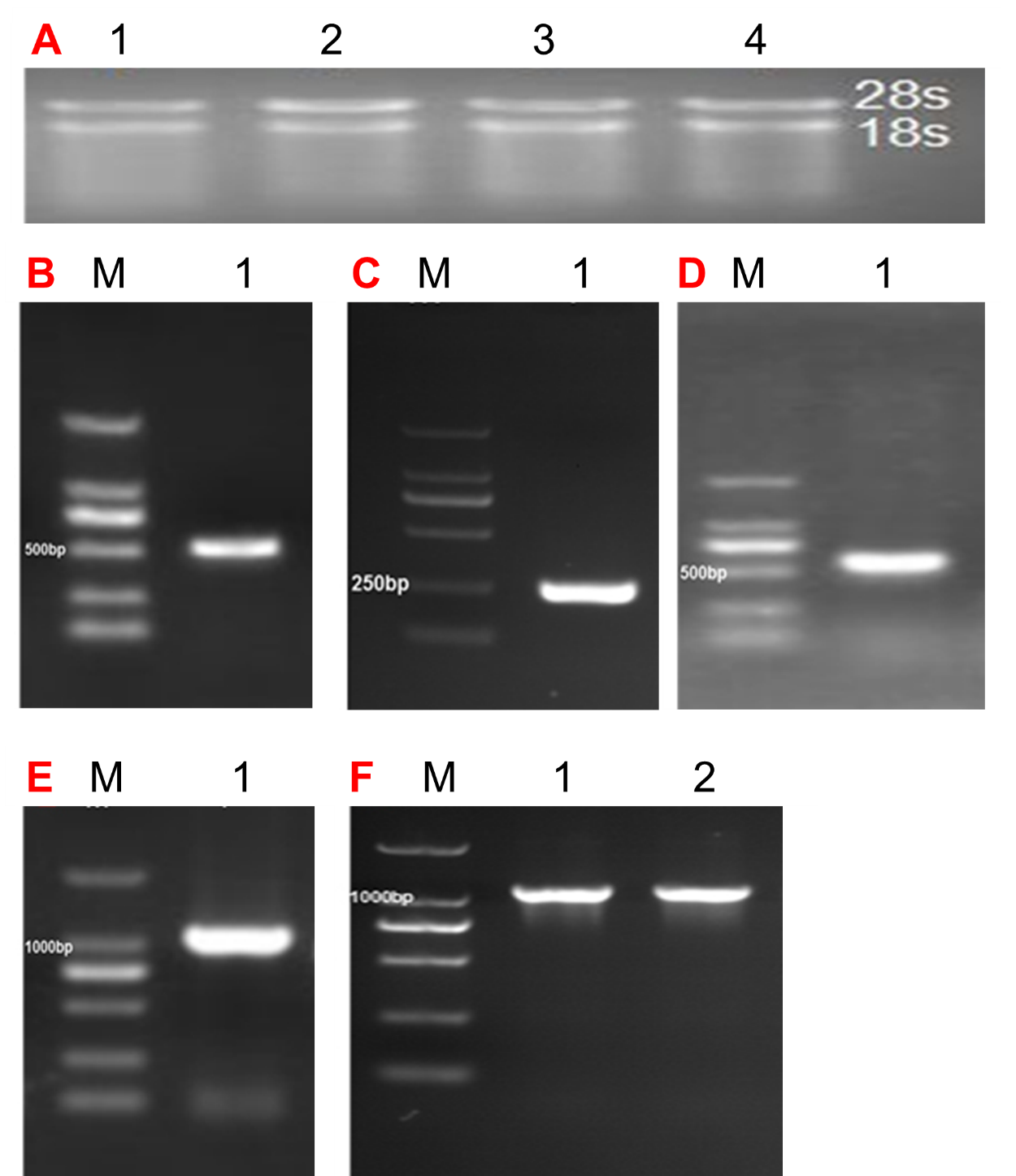
**

**Fig. S1** Profile of triticale rRNA and DNA in Agarose gels

**(A)** Agarose gels showing triticale rRNA and total RNA. **(B)** Northern blot of the intermediate sequence. **(C, D)** Northern blots of the fragments amplified using **(C)** 5' rapid amplification of cDNA ends (RACE) and **(D)** 3' RACE. **(E)** PCR amplification of the full-length triticale *TwNAC01* cDNA sequence. **(F)** PCR detection of positive clones from *Escherichia coli* TOP10 cells. Lane M in all gels: DL2000 DNA molecular size ladder showing 100, 250, 500, 750, 1,000, and 2,000 bp fragments (Takara Bio Inc., Da Lian, China).

**
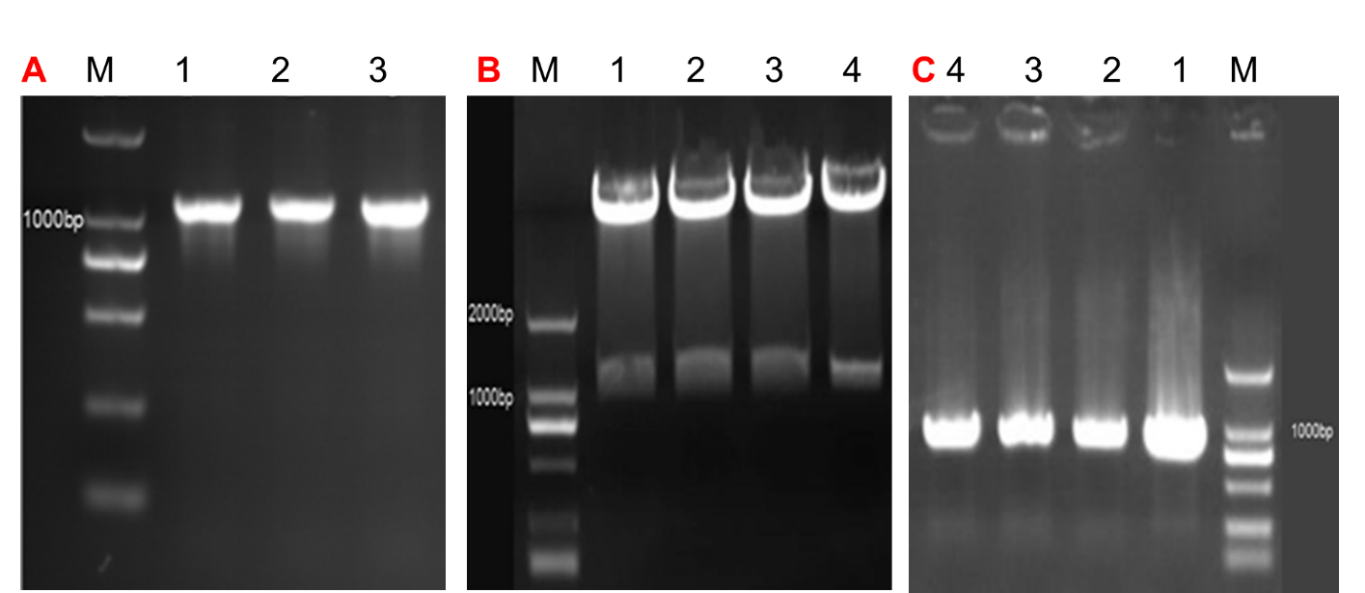
**

**Fig. S2** *TwNAC01* profile in transgenic *Arabidopsis thaliana*

A. PCR detection of the expression vector in the *E. coli* bacterial solution. B. Enzyme digestion of the recombinant plasmid pCAMBIA1300-35S, showing that the recombinant expression plasmid containing the target gene had been successfully constructed. C. PCR analysis of bacterial solution containing *Agrobacterium* lines carrying the recombinant plasmid pCAMBIA1300-35S-TwANC01. Lane M in all gels: DL2000 DNA molecular size ladder showing 100, 250, 500, 750, 1,000, and 2,000 bp fragments (Takara Bio Inc., Da Lian, China).

**
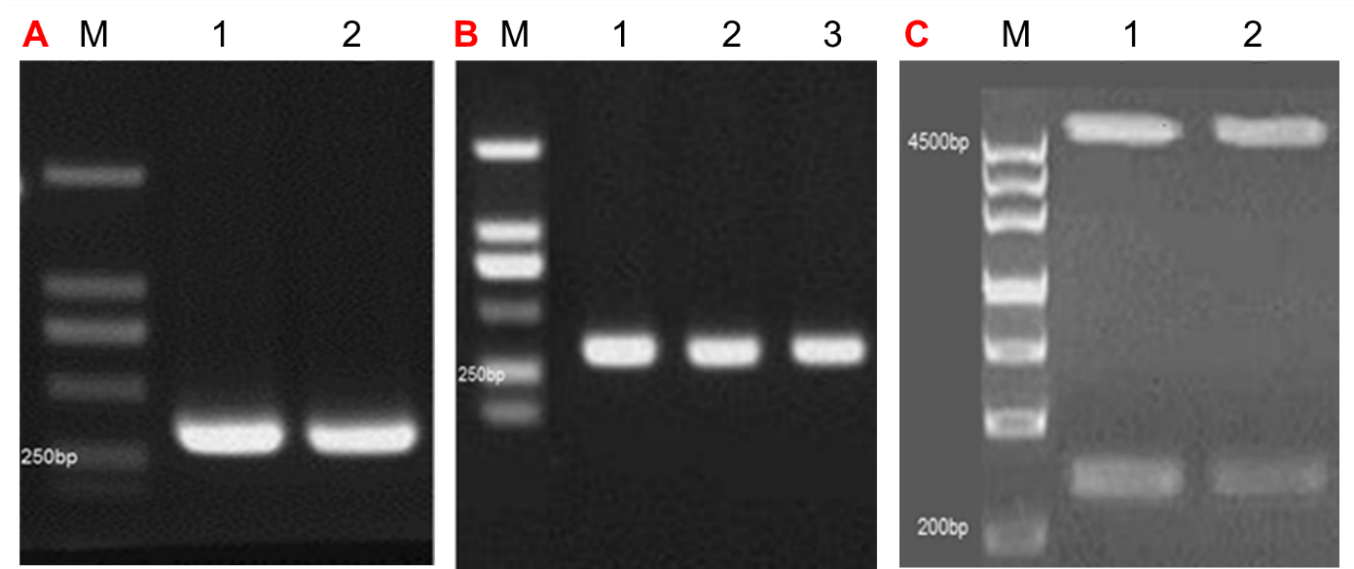
**

**Fig. S3** Profile of *TwNAC01* gene for silencing

A. PCR amplification of the *TwNAC01* gene fragment; Lane M, DL2000 ladder. B. PCR amplification of the *E. coli* bacterial solution showing positive clones; Lane M, DL2000 ladder. C. Recombinant vector digestion of the triticale *TwNAC01* gene fragment; Lane M, Marker III showing 4,500, 3,000, 2,000, 1,000, 800, 500, and 200 bp fragments.
